# Influence of Titanium Oxide Pillar Array Nanometric Structures and Ultraviolet Irradiation on the Properties of the Surface of Dental Implants—A Pilot Study

**DOI:** 10.3390/nano9101458

**Published:** 2019-10-14

**Authors:** Juan-Rey Leon-Ramos, Jose-Maria Diosdado-Cano, Carmen López-Santos, Angel Barranco, Daniel Torres-Lagares, María-Ángeles Serrera-Figallo

**Affiliations:** 1 Institute of Materials Science of Seville, CSIC-University of Seville, Américo Vespucio Street n 49, 41092 Seville, Spain; maserrera@ono.com (J.-R.L.-R.); mclopez@icmse.csic.es (C.L.-S.); angel.barranco@csic.es (A.B.); 2 Faculty of Dentistry, University of Seville, Avicena Street, 41009 Seville, Spain; xemita_879@hotmail.com; 3 Department of Atomic, Molecular and Nuclear Physics, Faculty of Physics, University of Seville, Reina Mercedes Street, 41012 Seville, Spain

**Keywords:** titanium oxide, dental implant, ultraviolet irradiation, surface

## Abstract

Aim: Titanium implants are commonly used as replacement therapy for lost teeth and much current research is focusing on the improvement of the chemical and physical properties of their surfaces in order to improve the osseointegration process. TiO_2_, when it is deposited in the form of pillar array nanometric structures, has photocatalytic properties and wet surface control, which, together with UV irradiation, provide it with superhydrophilic surfaces, which may be of interest for improving cell adhesion on the peri-implant surface. In this article, we address the influence of this type of surface treatment on type IV and type V titanium discs on their surface energy and cell growth on them. Materials and methods: Samples from titanium rods used for making dental implants were used. There were two types of samples: grade IV and grade V. In turn, within each grade, two types of samples were differentiated: untreated and treated with sand blasting and subjected to double acid etching. Synthesis of the film consisting of titanium oxide pillar array structures was carried out using plasma-enhanced chemical vapor deposition equipment. The plasma was generated in a quartz vessel by an external SLAN-1 microwave source with a frequency of 2.45 GHz. Five specimens from each group were used (40 discs in total). On the surfaces to be studied, the following determinations were carried out: (a) X-ray photoelectron spectroscopy, (b) scanning electron microscopy, (c) energy dispersive X-ray spectroscopy, (d) profilometry, (e) contact angle measurement or surface wettability, (f) progression of contact angle on applying ultraviolet irradiation, and (g) a biocompatibility test and cytotoxicity with cell cultures. Results: The application of ultraviolet light decreased the hydrophobicity of all the surfaces studied, although it did so to a greater extent on the surfaces with the studied modification applied, this being more evident in samples manufactured in grade V titanium. In samples made in grade IV titanium, this difference was less evident, and even in the sample manufactured with grade IV and SLA treatment, the application of the nanometric modification of the surface made the surface optically less active. Regarding cell growth, all the surfaces studied, grouped in relation to the presence or not of the nanometric treatment, showed similar growth. Conclusions. Treatment of titanium oxide surfaces with ultraviolet irradiation made them change temporarily into superhydrophilic ones, which confirms that their biocompatibility could be improved in this way, or at least be maintained.

## 1. Introduction

Titanium implants are commonly used as replacement therapy for lost teeth and much current research is focusing on the improvement of the chemical and physical properties of their surfaces in order to improve osseointegration [1,2].

Nanostructured surfaces are those less than 100 nm in at least one dimension. Ordinary techniques for surface treatment in implantology, such as acid etching (single or double, with different acids) or the creation of subtraction surfaces by bombarding particles of different size [3] have been replaced with more advanced techniques that enable the improvement of the nanometric structural morphology and the hydrophilicity of the titanium surface. Among the different techniques are laser-based, [4] thermal oxidative, [5] and electrochemical techniques, [6] resulting in multiple surface modifications (titanium-based nanotubes, [7] peptides, [8] multifunctional dendritic, [9] among others). Sol-gel coatings of titanium oxide (TiO_2_) are characterized by showing nanopores on their surface, which have demonstrated that they improve the biocompatibility of implants in animal and human models and lead to an improvement in the initial stages of osseointegration compared to other surfaces [10,11,12,13,14,15,16,17,18,19].

Titanium oxide surfaces can also incorporate strontium and phosphates to improve even further their capability for osseointegration [20,21]. Furthermore, there are studies that support a normal neuronal response (nerve conduction velocity, response to heat, amplitude and duration of the response) for these surfaces compared to others [22].

This improvement has not only been demonstrated in histological findings, but also in late loading prospective clinical trials and even immediate loading ones, with high success rates [17,23,24,25].

The modification of the physical parameters of a surface has an important effect on cell and tissue growth on it. This is called mechanotransduction, and it is defined as the process of cell signal transduction in response to stimuli or mechanical characteristics (among which are to be found surface characteristics, such as roughness). Mechanotransduction converts mechanical stimulus into a chemical sequence using membrane distortion. This occurs through mechanosensitive ion channels which are found ubiquitously in the cell membrane. Such channels increase or diminish the ion flow when the cell membrane is stimulated mechanically.

Responses to mechanical stimuli have been studied in many types of cells and in numerous different organic systems (osteocytes, endothelial cells, muscle cells or fibroblasts). In non-specialized mechanosensitive cells, the cytoskeleton is the protagonist of mechanotransduction, such that in response to the mechanical load, a remodeling of the cytoskeleton elements takes place, following a consistent pattern of deformability in which pre-stress tension plays an essential stabilizing role [26,27,28,29,30].

Thus, forces and mechanical parameters play an important role in the organization, growth, maturation and function of live tissue. At the cell level, many biological responses to forces or external mechanical characteristics arise from two specialized types of microstructures: focal adhesions which connect cells to the extracellular matrix, and adhesive bonding, which keeps adjacent cells together. The transmission of forces from the cell exterior through the extracellular matrix and from intercellular contact seems to control the establishment or the disassembly of such adhesions and initiates a cascade of intracellular signals which compromise numerous cell behaviors [30,31].

In the field of implantology, this phenomenon is of crucial importance through its influence on how implant surfaces affect the growth of cells responsible for bone formation. On the other hand, there is a lacuna about whether optical properties of a material could also have an effect on said cell growth, like other properties of the material.

Among the surface parameters that affect the response of the host tissue are wettability, roughness and chemical composition [32,33,34]. These surfaces are interesting to improve cellular adhesion to the peri-implant surface [35,36,37,38].

The improvement in biocompatibility on TiO_2_ surfaces would be obtained thanks to the intrinsic nature of the material, its design and construction. Another advantage of TiO_2_ surfaces is the possibility of anchoring functional molecules and drugs into its surface, although this was not explored in this study [39,40].

In this article, we address the influence of surface treatment in type IV and type V titanium discs (deposition of TiO_2_ thin films) on cell growth on them. Moreover, the influence of the UV light irradiation of the surfaces on cell growth will be examined.

## 2. Materials and Methods

Samples were used from titanium rods employed in the making of dental implants (Oxtein, Madrid, Spain). There were two types of samples: grade IV and grade V. The grade IV ones presented a diameter of 1 cm and a thickness of 0.2 cm, whilst the grade V ones presented a diameter of 1.5 cm and a thickness of 0.2 cm. This difference in diameter had no influence on the result or performance of the experiment. The different characterizations of the disks were carried out in the center region to assure that this difference in diameter had no influence on the result or performance of the experiment.

**Figure 1:**
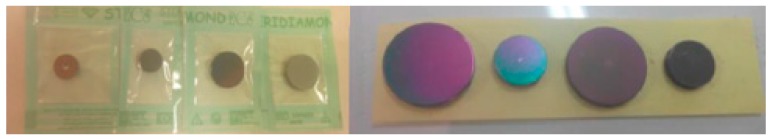
On the left are samples without a TiO_2_ film and on the right, samples with TiO_2_.

In turn, within each grade, two types of samples were differentiated, some untreated and others treated with sand blasting and subjected to double acid etching. This treatment is one of those approved and commonly used in dental implants placed in patients in dental practice. Oxtein^®^ implants have been subjected to surface modification procedures based on coarse-grain blasting (250–500 μm) and double acid passivation using a mixture of HCl with H_2_SO_4_ at a high temperature for several minutes. This procedure creates micro-wells superimposed on the surface of rough sandblasting, which results in characteristic micro-roughness [41]. Such surfaces, as mentioned above, are usually referred to in the literature as SLA. Thus, we obtained four types of sample which were sterilized using gamma rays and were packaged in individual envelopes, as shown in Figure 1. From these four original samples, another four were generated using a 250-nm thick nanocolumnar TiO_2_ film deposited using by Plasma-Enhanced Chemical Vapor Deposition (PECVD). Table 1 shows the nomenclature used for the eight samples.

The synthesis of titanium oxide film was carried out in a PECVD reactor. Plasma was generated in a quartz vessel by an external SLAN-1 2.45 GHz microwave plasma source (Plasma Consult, GmbH, Wuppertal, Germany). Preparation was carried out by applying power a MW power of 680 W with the sample holder at room temperature. The residual pressure of the chamber was <10^−5^ mbar. The deposition pressure was 7.5 × 10^−3^ mbar using pure O_2_ as plasma gas. The precursor used was titanium tetraisopropoxide dosed inside the chamber using a 16-outlet spray to distribute it homogeneously. The precursor line was kept at 50–70 °C to avoid condensation and 250 nm nanocolumnar films of titanium oxide were deposited on the titanium disk and substrates for specific characterizations. Additional details of the deposition system can be founded elsewhere [42,43,44].

Five discs of each of the configurations were made (in total, 40 discs). Thus, one of the discs was used to study X-ray photoelectron spectroscopy (XPS) and scanning electron microscopy (SEM). A second disc was used for the study of energy dispersive X-ray spectroscopy. A third disc was used for the study of profilometry and a fourth disc for the study of contact angle measurement or surface wettability and its progression of the contact angle on applying UV irradiation. Finally, a fifth disc was used for the study of biocompatibility and a cytotoxicity test with cell cultures.

### 2.1. Physical Typification Studies.

#### 2.1.1. X-Ray Photoelectron Spectroscopy (XPS)

An ESCALAB 210 (VG Scientific) spectrometer was used to determine the surface concentration of the samples by X-ray photoelectron spectroscopy (XPS). The spectrometer operated at a constant pass energy of 20 eV using monochromatic radiation Mg Kα (1253.6 eV). Surface concentrations in atomic percentages were determined quantitatively from the area of the main photoemission peaks. The peaks were corrected by the electron escape depth, the spectrometer transmission, and the photo-electron cross sections. Gaussian–Lorentzian functions were employed to determine the components of the photoelectron peaks using the CasaXPS program (Casa Software Ltd., Teignmouth, United Kingdom).

#### 2.1.2. Scanning Electron Microscopy (SEM)

The microstructure of the films was determined using a Hitachi S4800 SEM-FEG (Hitachi, Japan). The cross-section of a set of reference films grown on Silicon (100) were precisely determined by cleaving coated wafers.

#### 2.1.3. Energy Dispersive X-ray Spectroscopy

This technique is semi-quantitative and presents a greater accuracy when determining the composition of heavy elements than for light elements, therefore determining a more exact quantification for titanium than for carbon and oxygen. It is less precise than XPS and penetrates deeper into the sample, at 1 μm, presenting a sensitivity factor of 3%. The equipment employed was a Hitachi S4800 (Hitachi, Japan). The P/B ZAF (standardless) quantification method was used.

#### 2.1.4. Profilometry

The equipment used to measure the surface profiles was a MAHR (Mahr GmbH, Esslingen am Neckar, Germany) perthometer model profilometer employing the MARSURF XR 20 (Mahr GmbH, Germany) program, which complies with ISO 5436 standard. The parameters determined were the arithmetic mean roughness value (Ra), the root mean square (Rq), and the maximum depth of roughness (Rz). 

### 2.2. Biological Valuation Studies

#### 2.2.1. Contact Angle Measurement or Surface Wettability

Methods based on the Young–Dupré equation, such as the contact angle measurement or surface wettability, are used to determine parameters such as surface free energy or surface tension, which is the measurement of cohesion energy on an interface and expresses the free energy to increase, in a unit of area, the contact between two distinct phases. To perform the measurements, a Dataphysics OCA 20 (DataPhysics Instruments GmbH, Filderstadt, Germany) goniometer, a high-resolution camera, a drop dispenser, and the Dataphysics OCA 20 program were used. Macroscopic drops of double-distilled water (pH 7) with a volume of 1 μL and diiodomethane with a volume of 3 μL were dispensed on the samples surfaces and measured using the static sessile drop method. The surface tension values of the water were σ_a_ = 72.8 mJ m^−2^, σ_a_^P^ = 51 mJ m^−2^, σ_a_^D^ = 21.8 mJ m^−2^ and of the diiodomethane, σ_b_ = 50.8 mJ m^−2^ σ_b_^P^ = 44.1 mJ m^−2^ σ_b_^D^ = 6.7 mJ m^−2^, where σ^D^, the dispersive component and σ^P^, the pole component of the surface tension, were taken from the bibliography [45,46].

#### 2.2.2. Evolution of the Contact Angle under UV Irradiation

A Xenon discharge lamp (150 W) with a total photon intensity of ~2 Wcm^−2^ without filters was used for the characterization of the water contact angles under illumination. The UV irradiation intensity was ~0.05 Wcm^−2^, determined with a photodiode (Solar Light Comp. (Glenside, PA, USA) PMA 2100). The time intervals for performing the contact angle measurement were defined between 30 and 180 s, carrying out the measurement with double-distilled water on completing each exposure period and representing accumulatively the total exposure time to the UV radiation source.

#### 2.2.3. Biocompatibility and Cytotoxicity Test with Cell Cultures

The stem cells (hASCs) employed in this project to study cytotoxicity and cell proliferation came from the adipose tissue of healthy adults and were between 10 and 12 μm. These hASCs cells were isolated from vessel stroma from human lipoaspirates using liposuction from healthy adult donors. This study had prior authorization from the Ethical Committee of the CCMI (Minimally Invasive Surgery Centre) in Cáceres, where said study was undertaken. The samples were washed twice with a phosphate buffered saline solution (PBS) and they were digested at 37 °C for 30 min with a type 1 collagen solution, 18 U/mL in PBS. After this digestion, the samples were washed with 10% fetal bovine serum (FBS) treated with a 160 mM solution of ammonium chloride suspended in a culture medium with 10% FBS; then, the cells were filtered through a 40 mm nylon mesh. The cells obtained were cultivated in cell flasks at 37 °C and 5% CO_2_ for 7 days.

To perform the experiment on the samples, a single cell line was used. The samples were subjected to prior sterilization using UV radiation. The flask surface was used as a control, as well as polystyrene discs as a second control. Once the cells had expanded, they were trypsinized and were re-suspended in culture medium (DMEM without phenol red + 10% FBS, 1% penicillin/streptomycin, 1% l-Glutamine) and were adjusted to a final concentration of 50,000 cells/mL. From this suspension 100 μL were added to each sample (5000 cells/sample) and were left in culture for 72 h, adding 20 μL of DDEM every 24 h to each sample.

After the 72-h pre-incubation of the cells, cell growth was analyzed at a temperature between 0 and 5 °C. On the titanium samples, 100 μL were inoculated with cells in suspension and 10 μL reagent CCK-8 [2-(2-methoxy-4-nitrophenyl)-3-(4-nitrophenyl)-5-(2,4-disulfophenyl) 2H-tetrazolium, monosodium salt], followed by incubation for 1–4 h in a humidifier (37 °C, 5% CO_2_), and finally, adding 10 μL at 1% *w/v* SDS or 0.1 M HCl (to protect the samples) and carrying out measurements of absorbance at 450 nm using Facscalibur Benton Dickinson (BD Biosciences, San Jose, CA, USA) equipment to calculate the number of cells which adhered and grew from the absorbance measurement. In every case, cell proliferation was observed after an incubation period of 48 h. Only one disc was cultivated for each study group, therefore, statistics are lacking in this section. However, the mean and standard deviation of both the number of adhered cells and the absorbance in the non-SI samples versus the SI samples were obtained. The objective of this comparison was to ensure, as far as possible, the biocompatibility of the modified samples with respect to the unmodified and previously tested samples in other experiments.

## 3. Results

### 3.1. Analysis of Surface Composition Using XPS Spectroscopy

**Figure 2:**
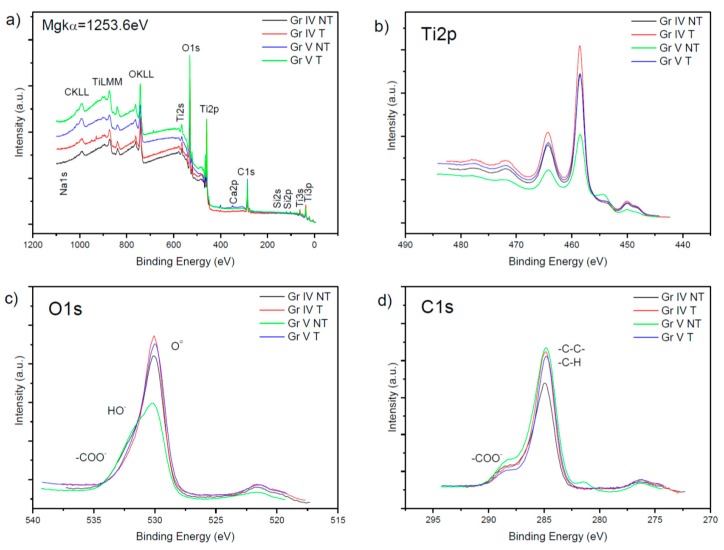
General spectrum, (**a**) of Ti2p regions, (**b**), O1s, (**c**), and C1s, (**d**) of untreated grade IV samples (black line) and treated (red line) and untreated grade V (green line) and treated (blue line) samples. (GR IV: Titanium grade IV; GR V: Titanium grade V; T: SLA treated; NT: SLA non-treated).

The Ti2p signal of TiO_2_ at 458.5 eV was used for calibrating the binding energy of the spectra. By observing chemical displacements in binding energies, we can confirm their state of oxidation and the species present. The first samples analyzed were the untreated grade IV samples (black line), grade IV treated (red line), grade V untreated (green line), and grade V treated (blue line). The spectra obtained are shown in Figure 2. In this figure, we can observe (a) the general spectrum for grade IV and V samples, where the presence of elements Na, C, Ti, O, Ca, and Si (with their corresponding main, secondary and Auger peaks) is evident in the grade IV samples and where, in the grade V ones, we can see the presence of Na, C, Ti, O, N, Ca, Si, S, P, and Al.

In section (b) of Figure 2, we can observe the 2p doublet of titanium, encountering the main peak, Ti2p3/2 at a binding energy of 458.5 eV (which indicates the presence of Ti^4+^); the secondary one at 462.5 eV and a satellite between 445 and 452 eV. In the untreated grade V sample, a peak was observed between 452 and 456 eV due to the presence of Ti–C bonds.

In section (c), we find the region corresponding to the 1 s peak for oxygen, with its highest energy peak at 530 eV corresponding to the Ti–O bond of titanium oxide and in the untreated grade V sample, we can see a shoulder in the main peak between 517.5 and 523 eV.

In section (d), we can see the region where the peak corresponding to C1s is, its main peak being at 284.9 eV due to the simple bonds C–C and C–H, and its satellite between 275 and 278 eV. Together with the main peak, a shoulder appears at 288.6 eV, which corresponds to the species –CCO–. Furthermore, in the untreated grade V sample, a signal can be seen between 280 and 285.5 eV, which could correspond to the species TiC [47].

**Figure 3:**
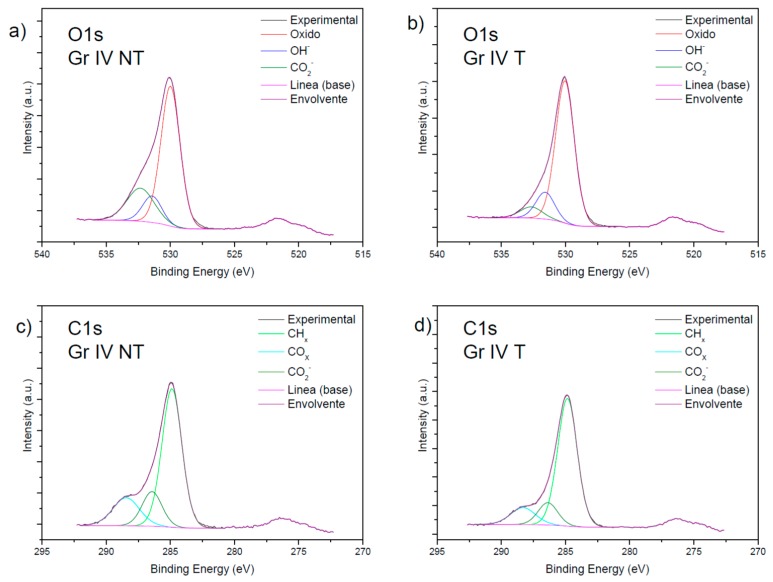
O 1s X-ray photoelectron spectroscopy (XPS) spectra of the untreated grade IV (**a**) and the treated grade IV surfaces (**b**). C1s spectra of the untreated grade IV (**c**), and the treated grade IV (**d**) surfaces. (GR IV: Titanium grade IV; GR V: Titanium grade V; T: SLA treated; NT: SLA non-treated).

In Figure 3, the species present in the oxygen and the carbon of the grade IV samples are studied. In sections (a) and (b), it is possible to observe the species formed by the oxygen in the untreated and treated grade IV sample, respectively, which are oxide (=O), hydroxide (–OH), and carboxylates (–COO–).

In sections (c) and (d), the species of C–H (hydrocarbons), –C=O (from ketones or aldehydes) and –COO– (carboxylates), formed by the carbon in the untreated and treated grade IV sample respectively, are shown.

**Figure 4:**
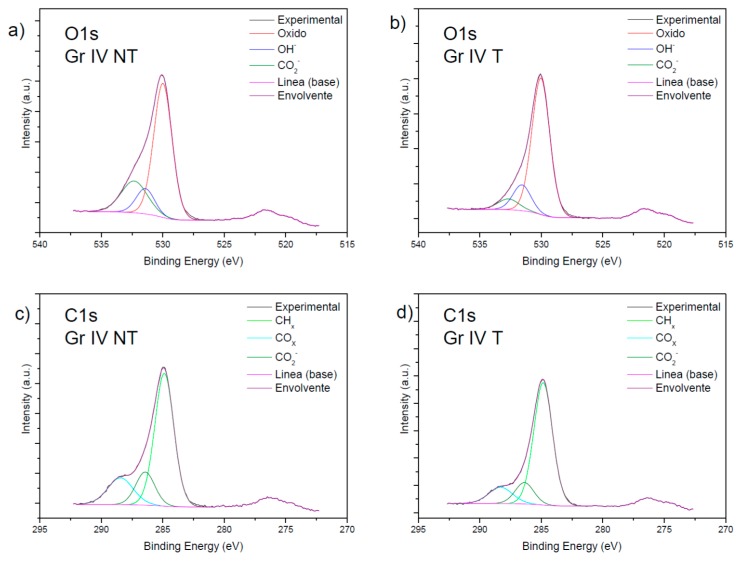
O 1s XPS spectra of the untreated grade V (**a**) and the treated grade V surfaces (**b**). C1s spectra of the untreated grade V (**c**) and the treated grade V (**d**) surfaces. (GR IV: Titanium grade IV; GR V: Titanium grade V; T: SLA treated; NT: SLA non-treated).

In Figure 4, the regions of oxygen and carbon, with the respective species present in them for the treated and untreated grade V samples, were studied. In sections (a) and (b), we see the oxygen region with species of oxides (O^−2^), hydroxides (OH^−^), and carboxylates (CO_2_^−^).

Sections (c) and (d) show the carbon region, where species of hydrocarbon (CH_X_), carbon oxides (CO_x_), carboxylates (CO_2_^−^) are observed and, only in sample c), titanium carbide (TiC), were studied. The presence of TiC was deduced by observing the new signals at 280–282.5 eV on the green line in section (d), and the signal at 452–456 eV on the green line in section (b).

Table 2 shows the concentrations of the elements present in the treated and untreated grade IV and V samples. It can be seen that the majority elements were Ti, O and C. On the other hand, a larger amount of oxygen can be seen in all the samples compared to titanium. The oxygen which did not form titanium oxide may have formed carbonates, acids, aldehydes, ketones or alcohols with carbon, which was present in large quantities. Al was present in the concentrations of grade V and silicon in those of grade IV.

With regard to the analysis of the surface composition using XPS spectroscopy in the samples with a film of TiO_2_, the elements that appeared were Ti, O and C. Moreover, in the treated samples, calcium appeared on the surface. A large amount of C can be seen on the surface and inside the layer deposited (Table 3).

**Figure 5:**
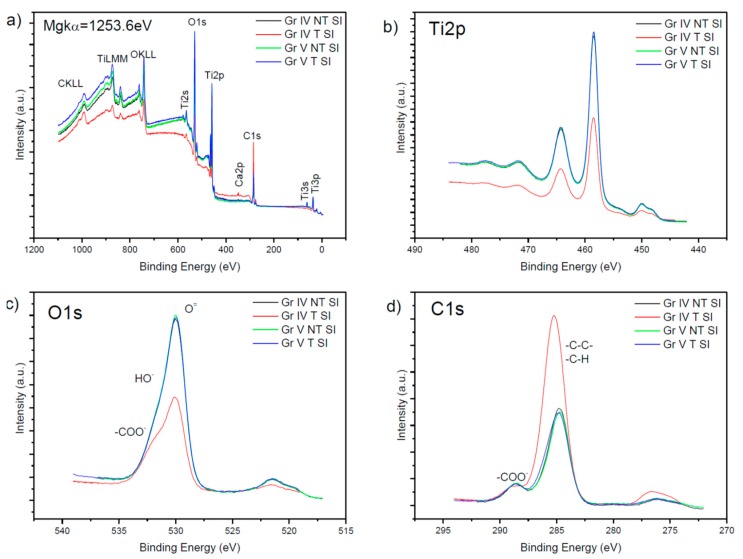
XPS survey spectra, (**a**), Ti 2p XPS spectra (**b**), O1s spectra (**c**), and C 1s spectra (**d**), of untreated TiO_2_ grade IV samples (black line), treated (red line), untreated grade V (green line) and treated (blue line) samples. (GR IV: Titanium grade IV; GR V: Titanium grade V; T: SLA treated; NT: SLA non-treated; SI: Treated with TiO_2_ film).

In Figure 5, the spectra of the grade IV and V sample are compared with TiO_2_. In section (a), the general spectrum with elements Ti, O, C, and Ca can be seen.

In section (b), the two 2p peaks of titanium can be seen, the main and most intense one being Ti2p3/2 at a binding energy of 458.5 eV, which indicates the presence of Ti^4+^, which is consistent with the layer of TiO_2_, and a secondary less intense one, Ti2p5/2, at 462.5 eV, which were half as intense as both the main peak and the secondary one in the grade IV T SI. Furthermore, a satellite can be seen between 452–455 eV.

Section (c) shows the region corresponding to the 1s oxygen peak with its highest energy peak at 530.8 eV corresponding to the Ti–O bond in titanium oxide and its respective satellites at 522.5 eV, the main peak in the grade IV T SI sample being half as intense, and we can also see a shoulder between 531 and 533 eV corresponding to double and simple bonds between oxygen and carbon, which is broader in this latter sample.

Finally, in section (d), we can see the region where the 1s carbon peak can be found, its main peak being at 284.9 eV and at 285.6 eV in the grade IV T SI sample and its satellites near to 277 eV, we can also see that the main peak of the grade IV T SI sample is twice as intense. Together with the main peak a shoulder appears at 288.6 eV in grade IV NT SI, which corresponds to the species –COO–.

Table 3 shows a significant amount of C in the form of C–H and O, combined with C. The O/Ti ratio is very similar in the treated and untreated grade V samples, however, in the grade IV samples, there is a greater difference between treated and untreated, and this ratio increases in the treated samples.

The C/Ti ratio increased in the treated samples, the treated grade IV sample being the one presenting a greater C/Ti ratio, and the untreated sample of the same grade in turn presented a C/Ti ratio higher than those in grade V. When comparing Table 2 and Table 3, a decrease of the O/Ti ratio is seen and in turn, there is an increase in the C/Ti ratio.

By comparing the original samples with those presenting a layer of titanium oxide, it is confirmed that the surface is equally composed of titanium, oxygen, and carbon. It can also be observed that the bands of these elements have relatively similar intensities to the original samples in the case of the grade IV samples, whereby the untreated one is more intense, and they vary compared to the intensity of the grade V samples. 

### 3.2. Surface Analysis of Samples by Scanning Electron Microscopy (SEM)

**Figure 6:**
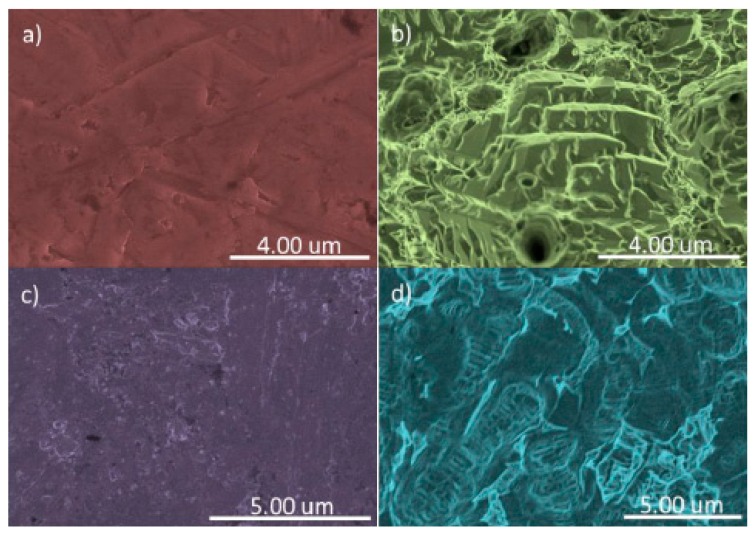
SEM images of the untreated with TiO_2_ film. (**a**) Grade IV non-SLA sample; (**b**) Treated (SLA) grade IV; (**c**) Untreated (non-SLA) grade V; (**d**) Treated (SLA) grade V.

**Figure 7:**
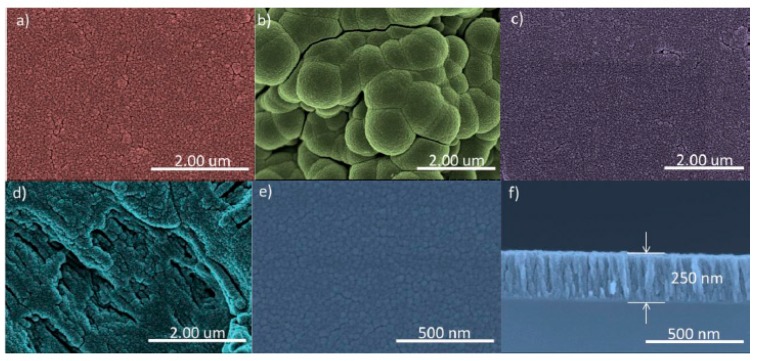
SEM images of samples treated with TiO_2_ film. (**a**) Untreated (non-SLA) grade IV; (**b**) treated (SLA) grade IV; (**c**) untreated (non-SLA) grade V; (**d**) treated (SLA) grade V; (**e**) and (**f**) frontal image and cross-section of the TiO_2_ film on a silicon plaque (100).

On the untreated grade IV samples, a homogeneous surface with low roughness was present, whereby no repeated patterns are seen on the surface and it presents typical machining defects, differing from the treated grade IV sample, where an image can be seen with greater surface roughness with unevenness, crests, and flat areas of the order of a micron (1–4) distributed evenly over the surface due to the double etching electrolytic treatment, patterns repeated across the surface. In the untreated grade V sample, a slightly rough and homogeneous structure can be seen, just as in the untreated grade IV sample, with characteristic machining marks and defects; at the same time, the treated grade V sample presents surface roughness in the form of small hillocks and hollows of around 500 nm uniformly distributed over the surface, these patterns repeating on the surface due to texturing treatment. Figure 6 and Figure 7 show the surfaces of the samples using SEM.

**Figure 8:**
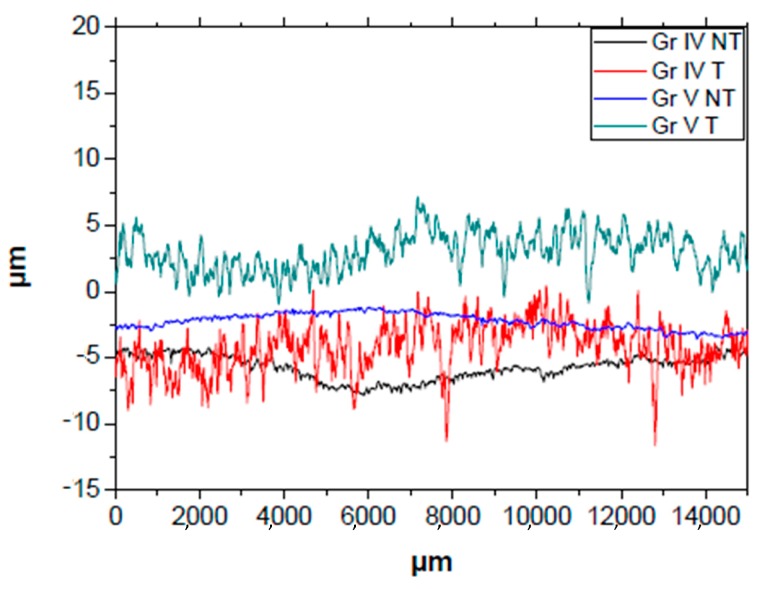
Roughness profile of the untreated with TiO_2_ samples. (GR IV: Titanium grade IV; GR V: Titanium grade V; T: SLA Treated; NT: SLA non-treated).

**Figure 9:**
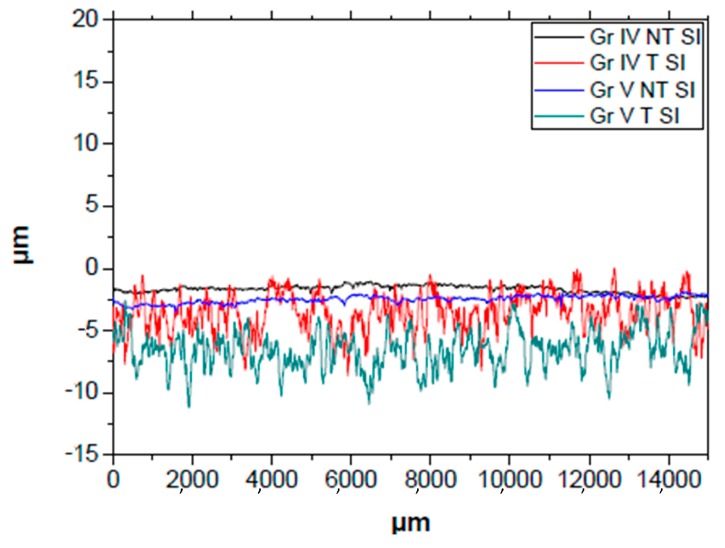
Roughness profile of the treated with TiO_2_ samples. (GR IV: Titanium grade IV; GR V: Titanium grade V; T: SLA treated; NT: SLA non-treated; SI: Treated with TiO_2_ film).

Compared to the surface analysis of the samples on which a nanocolumnar film of TiO_2_ was created using scanning electron microscopy (SEM), to calculate the thickness of these films of nanocolumnar titanium oxide, a reference sample of silicon was used. The cross-section of the silicon reference sample (100) can be seen and the thickness value of the film was obtained, which was around 250 nm. The grade IV NT SI and grade V NT SI samples presented a homogeneous surface (Table 4 and Table 5). The distribution of the pillars of titanium oxide over the whole surface can be seen. On the other hand, in the grade V NT SI sample, on the front and cross-section images of the TiO_2_ film on the silicon wafer (100), a clear difference in the morphology and the size of the film patterns can be observed. The grade IV T SI sample presents a new morphology with globular agglomerations of the pillar arrays of TiO_2_, distributed over the surface. These new patterns must be due to the initially treated surface of the sample favoring the growth over the troughs and crests of the sample. However, in the grade V T SI, the obtained image shows that the surface troughs and crests were accentuated compared to the grade V T, coating the troughs and crests of the original surface of the sample. Figure 7 shows the surfaces of the TiO_2_ samples using SEM. The roughness profiles can be seen in Figure 8 and Figure 9.

### 3.3. Characterization of the Composition of the Samples Using EDX

The atomic percentage of the sample elements can be seen using EDX analysis. It was observed that the majority component was titanium in all cases, its composition being considerably greater in the grade IV samples and in the treated samples. The species present in the highest proportion were oxygen, followed by carbon and aluminum. The atomic proportion of each sample is shown in Table 6.

Regarding the characterization of the composition of the samples modified by nanocolumnar TiO_2_ structures using EDX, we can see that the concentrations of the elements present values similar to the ones obtained in the untreated samples and slightly lower than the treated ones without deposition of the titanium oxide films. On the other hand, it can be seen that the concentrations are practically comparable with each other, presenting an atomic concentration of titanium of around 75%, both in the treated and in the untreated samples (except for grade IV T SI, which present 65% titanium and a larger amount of oxygen and carbon).

Comparing these results with the samples without a deposition of TiO_2_ revealed that aluminum was not present, the concentration of carbon diminished drastically in all cases, except grade IV T SI, and an increase in the concentration of oxygen occurred, presenting a slight decrease in the case of titanium. Table 7 reflects the atomic percentages of each sample of this type.

### 3.4. Surface Roughness Determined by Profilometry in the Samples

Table 4 shows the Ra (arithmetical mean roughness) values, Rq (root mean square roughness) and Rz (maximum roughness) of the original samples. By comparing the 26 samples of grade IV with those of grade V, we can see the mean roughness of the former was considerably higher, as well as the maximum roughness. By observing Figure 8, we find that the untreated grade IV sample (black line) and the untreated grade V sample (blue line) presented a similar roughness and much less than the treated samples.

Concerning the surface roughness determined by profilometry in the TiO_2_ samples (Table 5), and Figure 9, we can see the profiles of the four samples with the titanium oxide film presenting more fluctuations in maximum and minimum values of the roughness profile in the treated grade IV and V, just as in the samples with no TiO_2_ coating. By comparing the roughness presented in Table 5, a greater maximum roughness and a mean roughness can be seen in the treated samples compared to the initially untreated ones, as in the case of Table 4. Furthermore, by adding the titanium oxide coating, the Ra, Rz, and Rq roughness values were of the same order of magnitude as the original samples, presenting almost identical values in the grade IV and grade V samples. The grade IV NT SI and grade IV T SI samples produced a decrease in Ra and Rq of between 90 and 100 nm, 20 nm in grade V NT SI and an increase of these values by 70 nm in grade V T SI. On the other hand, Rz decreased by 0.090 μm and 3.750 μm in grade IV NT SI and grade IV T SI, respectively, and an increase of 0.050 μm and 0.310 μm in grade V NT SI and grade V T SI.

### 3.5. Measurement of Surface Energy and Wettability of the Samples

Table 8 shows the total surface tension and their two components, dispersive and polar, contact angles and roughness factor. It can be seen that the surface tension of the treated grade IV sample was much greater than the other tensions, the untreated grade IV sample being the one with the lowest surface tension. The grade V samples presented a similar surface tension to each other (37.8 and 39.4 mN/m). On the other hand, it can be seen that the water contact angles were greater in the treated grade IV and V samples compared to the untreated ones. Regarding diiodomethane contact angles, the grade IV T sample presents the lowest angle (more oleophilic behavior) and the grade V T sample the greatest angle (less oleophilic), presenting a similar value in the case of the untreated ones of both grades. On the other hand, it can be seen that the Rw roughness factor increased in the treated sample, as did its ideal water contact angle.

The untreated samples presented hydrophilic surfaces and were less oleophilic than the treated ones. Furthermore, the Rw roughness factor and the contact angle were present, involving a totally flat surface without roughness.

**Figure 10:**
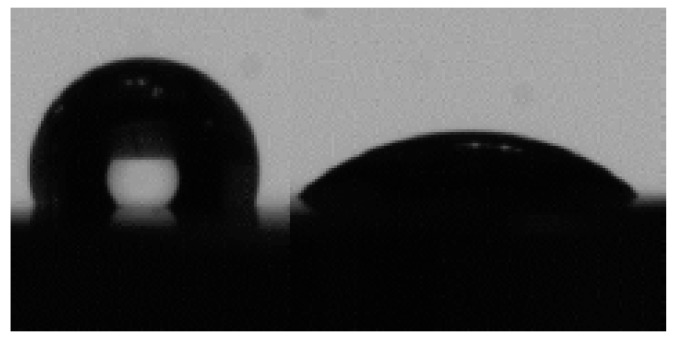
Image of the wettability of the grade IV NT SI sample with a drop of water (left) and with a drop of diiodomethane (right) on its surface.

Regarding the measurement of surface energy and the wettability of the nanocolumnar TiO_2_ treated samples, in Figure 10, we can see a drop of water (left) and a drop of diiodomethane (right) on the grade IV TiO_2_ treated sample, observing the hydrophobic behavior with a water contact angle greater than 90° (106.7°). This value, as well as the surface tension, increased compared to the same untreated sample. The drop of diiodomethane presented a low contact angle (43.6°), corresponding to an oleophilic behavior and remaining constant compared to the sample with no titanium oxide film. Surface tension enhancement was accompanied by an increase of its polar component with the TiO_2_ coating. However, the dispersive component only increased for the grade V T SI, which led to the greatest surface tension.

The grade IV T SI sample is a special case which presented a lower surface tension than the grade V T SI sample, which may be due to the greater concentration of surface carbon. On the other hand, the contact angle or the wetting of the surface both with water and diiodomethane from which the surface tension was determined is shown. It can be seen that the samples with prior treatment both the grade IV and the grade V ones were much more hydrophobic and less oleophilic than the ones that did not present initial treatment. On comparing the roughness factor and ideal angle values, the ratio between roughness and contact angles can be seen, confirming the increase in hydrophobicity by the addition of the titanium oxide film, thus increasing roughness (Table 9).

### 3.6. Study of Photoactivity of the Sample Surfaces Using UV Irradiation

A decrease in the water contact angle was observed over time by irradiating the samples, with UV irradiation of 0.038–0.048 Wcm^−2^.

**Figure 11:**
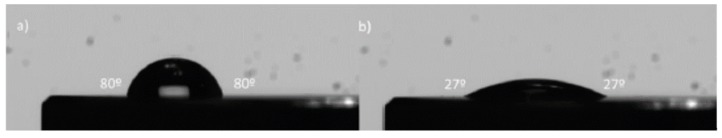
Image of the grade IV NT with a drop of water before (**a**) and after exposure to UV irradiation (**b**).

**Figure 12:**
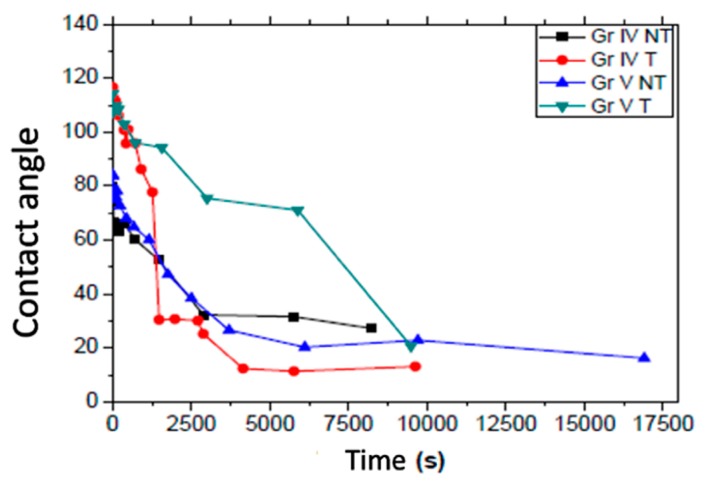
Progression of water contact angle regarding UV irradiation time on the grade IV and grade V original samples.

In Figure 11, we can see, on the left, an image showing (a) the initial grade IV T sample with its water contact angle and (b) a decreased water contact angle after the application of UV irradiation to the surface for 8298 s. In Table 10, we can see the initial contact angles of the non-irradiated samples and the final angles of the samples after irradiation with UV light. The UV irradiation time counted until the water contact angles remained constant (Figure 12).

Initially, the treated samples were much more hydrophobic than the untreated ones, both in grade IV and in grade V. Nevertheless, the water contact angle of the grade IV samples decreased much more rapidly after applying UV light. All the samples presented a gradual decrease in the contact angle to the superhydrophilic values of the contact angle by application of ultraviolet irradiation on the surface of the samples at small intervals of exponential time (30–180 s). Figure 12 shows that all the samples became hydrophilic after accumulating two hours of UV exposure. In addition, the treated samples presented greater initial hydrophobic behavior compared to the untreated ones, and furthermore, a sharper decrease of the contact angle, much higher for the treated grade IV sample compared to the treated grade V one.

**Figure 13:**
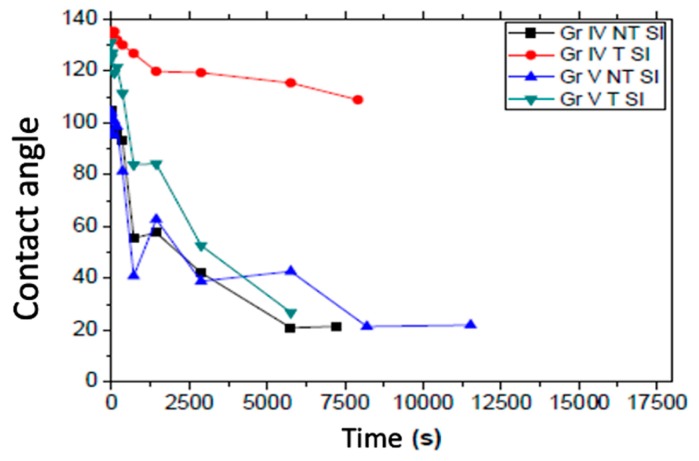
Progression of water contact angle regarding UV irradiation time on the grade IV and grade V samples with TiO_2_ film (SI).

Regarding the study of the photoactivity of the surfaces modified with nanocolumns of TiO_2_ by UV irradiation (Figure 13, Table 11), in Table 11, we can see the initial and final water contact angles of the samples with the deposition of TiO_2_, the surfaces with prior roughness (treated) being again more hydrophobic and the initial contact angle increasing in all cases. A significant decrease in the contact angle after UV irradiation can be seen in all the samples except the SI treated grade IV sample, which presents a more gradual decrease in the contact angle over time as can be seen in Figure 13. As occurred with the original samples in Figure 12, when comparing the samples with each other, it can be observed that not all the samples presented the same speed in the hydrophilic photoactivation, since the grade IV T SI and the grade V T SI samples start from higher angles than grade IV NT SI and grade V NT SI. Furthermore, the reduction in the water contact angle in grade IV NT SI and grade V NT SI presented the same rate, whereas the rate in the grade V T SI sample was slightly higher, unlike the grade V T, with the higher rate (Figure 12).

### 3.7. Study of the Results of the Sample Cell Cultures

**Figure 14:**
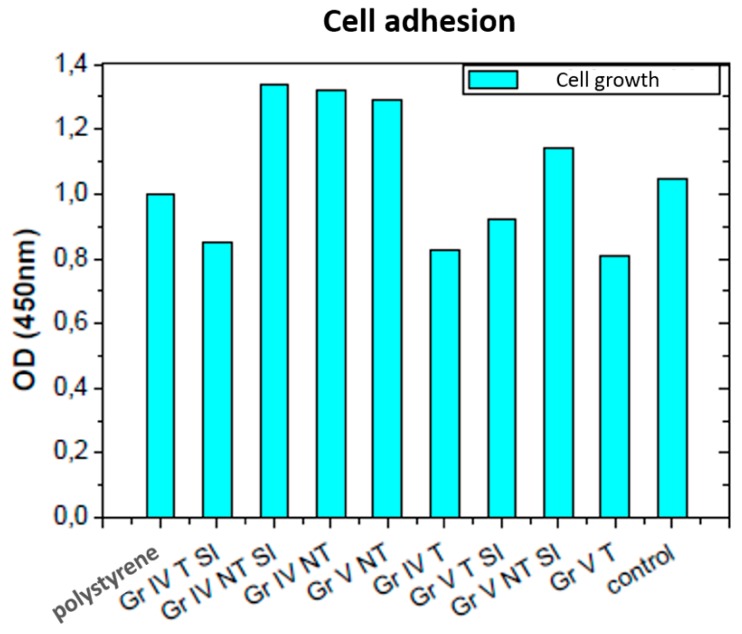
Graph of the results of culture of mother cells on different samples.

**Figure 15:**
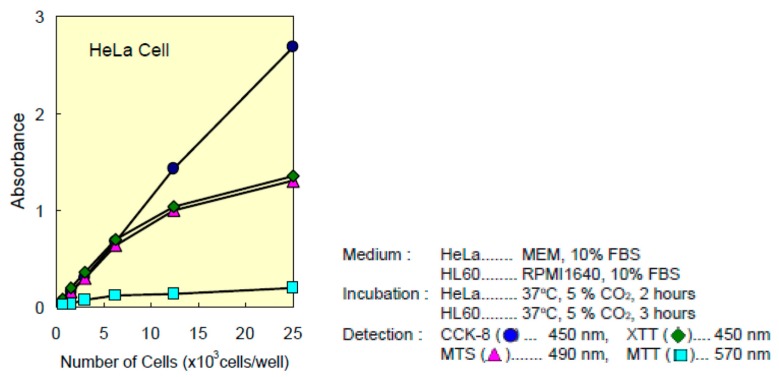
Representation of the number of HeLa cells with regard to absorbance. It is found that 1 OD are 8.93 × 10^3^ HeLa cells (blue circle). This has been the relationship we have used to extrapolate the absorbance obtained with the number of cells present.

Figure 14 shows polystyrene (growth reference), the original samples and with titanium oxide and the second control (surface of the culture flask). Polystyrene is a plastic used as a positive control. All the samples were biocompatible and enabled cell growth on them, the greatest growth being on the sample with the grade IV NT SI titanium oxide film, followed by the grade IV NT, which presented very similar growth patterns. The grade IV T SI sample presented a slight increase in growth compared to the grade IV T. However, the grade V NT sample had higher growth than the sample with the titanium oxide film, grade V NT SI. Finally, the grade V T SI sample improved its growth in comparison with the original grade V T sample, as can be seen in Table 12, where the absorbance values obtained in Figure 14 are presented, and the corresponding number of adhered cells obtained by interpolation from the HeLa cell graph in Figure 15.

It can be seen that the grade IV samples presented similar behavior, with a significant improvement in the ones that presented the titanium oxide film, and in the grade V samples, the original untreated one presented a better behavior and the one that presented a film of titanium oxide in the case of the treated one.

From this, we can deduce that the untreated grade IV titanium samples are the ones that presented the best cell growth, this being greater in the samples containing the titanium dioxide film. The untreated grade V sample is the one that occupies the third place regarding growth, followed by the grade V sample not treated with titanium dioxide.

The fifth place for growth is awarded to the control group, followed by the treated titanium V sample coated with titanium dioxide. The treated grade IV samples come next, where the sample coated with titanium dioxide had slightly larger growth. The sample with least growth was the treated grade V one.

Therefore, only the grade IV NT, grade IV NT SI, grade V NT, and grade V NT SI presented more growth than the control sample. In these samples, only in the untreated grade IV ones did cell growth improve by adding a layer of titanium dioxide.

The treated grade IV and V samples had less growth than the control sample. Hence, it can be deduced that sand blasting treatment and double acid etching did not have a beneficial effect when it comes to increasing cell growth, although cell proliferation improved on addition of titanium dioxide to these samples.

The absorbance for the group of samples without modification SI was 1.04 ± 0.28 compared to 1.05 ± 0.20 in the group of samples with said modification (*p* = 0.989; Mann–Whitney U). Regarding the number of adhered cells, this was 9.34 × 10^3^ ± 2.49 × 10^3^ in the sample group are SI modification, compared to 9.37 × 10^3^ ± 1.85 × 10^3^ in the group with SI modification (*p* = 0.988; U of Mann–Whitney) Therefore, we can deduce, with the limitations of our study, that the biocompatibility of the samples was not altered by our modification.

## 4. Discussion

### 4.1. Analysis of the Surface Composition of the Samples Using XPS

The majority elements in all the samples were Ti, O, and C. In the grade V samples, Al was present, probably because of the shot-blasting applied to them. The grade IV samples contained Si, probably due to the presence of grease and lubricants during their machining.

The untreated samples contained the largest O/Ti percentage. From the results, it can be deduced that there was more Ti in the grade IV T samples and less O and C than in the untreated ones and that in the grade V T sample, there was more Ti and O than in the untreated ones. The grade IV T SI and grade V T SI samples contained Ca on their surfaces, as well as more C. The O/Ti ratio was similar in the grade V T SI and grade V NT SI, whilst it was greater in the grade IV T SI sample than in the grade IV NT SI sample.

### 4.2. Surface Analysis Using Scanning Electron Microscopy of the Original Samples

In the same way as there are mechanized and passivated dental implants, the untreated samples presented a low roughness, obtained from the cutting of the metal, and in the treated samples, an increase in surface roughness through the nanostructure.

In the grade IV T SI sample, globular clusters of TiO_2_ can be seen, whilst in the grade V T SI, more valleys and crests are seen than on the original sample, encountering a nanocolumnar TiO_2_ surface on both samples, as in other studies [2,48]. The surface of the grade NT SI is homogeneous. The nanostructure morphology presented in the grade IV NT SI and grade V NT samples is similar to the film of TiO_2_ seen in other studies [14], presenting a well-defined 500 nm-thick nanocolumnar microstructure and well-defined pores with an average thickness of 30 nm. The formation of nanocolumnar titanium coatings evenly across the whole surface is evident with different morphologies depending on the initial sample, with a thickness of 250 nm equivalent to flat silicon (100), which can be extrapolated to nanostructured surfaces achieved in other studies [49]. Some authors have shown that when a nanocolumnar morphology is formed using an anodizing process of a TiO2 surface, protein absorption and cell adhesion improve [12,14].

### 4.3. Characteristics of the Composition of the Samples Using EDX

On all the samples, Ti was the majority element, followed by O, C, and Al, which were higher in the treated samples.

Comparing these results with the samples with TiO_2_ deposition reveals that aluminum was not present, the concentration of carbon decreased drastically in all cases, except for grade IV T SI, and an increase in the concentration of oxygen took place, as occurs in other studies [11], presenting a slight decrease in the case of titanium. By comparing the results, it can be deduced that the film presented less species of carbon and the layer of titanium oxide was conformal.

### 4.4. Surface Roughness of the Samples Determined by Profilometry

Treated grade IV and V samples with and without TiO_2_ coating presented a higher surface roughness value (Ra, Rq y Rz) in all cases, presenting minor variations between those deposited and those not deposited with titanium oxide. The nanostructure on the sample surfaces with TiO_2_ seen previously in scanning electron microscopy would be a positive quality in the increase in removal torque through an improvement in bone deposition, which leads to better bone implantation of the dental implant [50].

### 4.5. Measurement of Surface Energy and Wettability of the Original Samples

The surface tension and wettability values match those of other similar studies [36], although the angles formed between diiodomethane and the treated grade IV and V samples were considerably lower. On the other hand, the influence of the roughness on the contact angle was confirmed by increasing its value for the samples with a greater Rw. Surface tension and the contact angle increased more in the treated samples compared to the untreated ones, the treated samples being more hydrophobic and less oleophilic.

In the samples treated with a nanocolumnar titanium oxide coating, the surface tension and water contact angle values increased. 

### 4.6. Study of Photoactivity Surfaces Using UV Irradiation

It was observed that the water contact angle decreased in all the samples after accumulating two hours of UV light exposition. Furthermore, it can be seen that the treated samples presented a better initial hydrophobic behavior compared to the untreated ones. A sharper decrease in the contact angle was noted, which was higher in the treated grade IV sample, which could indicate greater crystallinity or order compared to the grade V T one, the latter presenting a more amorphous surface [51]. The untreated grade IV and V samples presented an intermediate decrease in the contact angle, slower than in the treated grade IV one and faster than in the treated grade V one. This decrease was very similar in both samples, which presented a similar morphology to the SEM images.

The samples with titanium oxide coating presented a greater initial contact angle than the original samples and at the same time, the speed of decrease of the contact angle was slower.

In the other TiO_2_ films published and studied in the bibliography, a decrease was observed in the contact angle after applying UV irradiation (under visible light of wavelengths lower than 400 nm), turning the superficial film hydrophilic [52].

The slower decrease in the contact angle is associated with amorphous titanium, slightly crystalline structures of very thin layers. The decrease in the contact angle was greater in samples with anatase and rutile titanium oxide. On the other hand, the decrease in the contact angle reverted after a certain time, depending on the crystallinity of the film [53].

It must be highlighted that the degree of hydrophilicity achieved using UV irradiation depended on the amount of anatase that the surface contains, this factor increasing with it [28].

One way of ensuring that a nanocrystalline structure is achieved is to subject the TiO_2_ surfaces to an anodizing process [2].

On comparing all the measurements, a clear decrease in the contact angle can be seen on activated the titanium oxide surface with UV light after 90–120 min. This decrease was not as significant in the grade IV sample with treatment and a titanium oxide film due to an excess of carbon, which may be due to contamination. These wettability control properties enable the surface to be modified and to use the ideal conditions for cell proliferation (improving plasma adherence to the surface), although surfaces become hydrophobic again with aging [36].

### 4.7. Study of the Results of the Sample Cell Cultures

All the samples induced cell growth, that is, they were biocompatible. Although the samples did not show the same disc diameter in all cases, we believe that this limitation did not affect the results of the study, since the number of cells sown was similar in all the discs, keeping the drop away from the edge of the disc in all the cases, even in the smallest samples. Nanomaterials are structures with a size between 1 and 100 nm. This improves the electrical, thermal and ion conductivity properties, together with catalytic activity. A nanostructured surface increases cell growth and reduces unfavorable immunological reactions.

All the samples coated with pillar array titanium oxide structures presented greater growth than the original samples, except for the grade V NT SI. Hence, the reason why the grade V NT SI did not increase cell growth compared to the original sample will have to be investigated, since the improvement in biocompatibility on TiO_2_ surfaces compared to other surfaces has been well demonstrated [11,13,14,15,54,55,56].

The importance of TiO_2_ is not only the amount of cells that grow or deposit on it, but also the genetic modifications that are induced in them. It has been ascertained that by comparing groups of titanium oxide with control groups, there was biocompatibility in both, although there were no significant differences in cell growth, but there were in the expression of some genes relating to bone formation, these being greater in the Ti oxide surfaces [26].

Previous studies have stated that long nanotubes were more efficient than short ones in bone marrow stem cell differentiation regarding proliferation, differentiation, immune response and inhibition of genes responsible for cell adhesion [12]. There is controversy on this point since some authors who concluded that nanotubes of 70 nm in diameter are optimum for the development of human stem cells. On the other hand, some authors stated that diameters of 30 nm induced less stress in osteogenic cells than those measuring 70 nm [28].

ATP1A2 and MAP3K11 regulating genes have also been studied, related with the transport of Na and K, and how titanium oxide can induce their expression. If we compare nanotubes 30 nm in diameter (anodized at 5 V) and 100 nm (anodized at 20 V) on surfaces with anodized titanium oxide, the larger nanotubes induced greater expression of the OPN gene, regulating 144 genes related with osteogenesis, whilst smaller nanotubules only regulated 30 genes involved in this function [30]. Lavenus et al., on the other hand, stated that 1-nm diameter nanotubes induced a greater cell differentiation in human mesenchymal stem cells [31].

Also important is the way in which cell differentiation occurs when we use this type of nanometric surfaces. Some authors have managed to differentiate stem cells into endothelial cells from surfaces modified with titanium oxide by comparing nanotubular TiO_2_ samples with TiO_2_ samples associated with nano and microroughness. These studies stated that the rates of cell differentiation were better when the surfaces were associated with nano and microroughness [27]. This might explain why the grade V NT sample showed a greater growth than the grade V NT SI one, due to the fact that the grade V NT sample is the only one which presented a greater microroughness before treatment with titanium dioxide.

In other tests on bone marrow stem cell differentiation on surfaces coated with titanium oxide, authors used titanium oxide with rutile with a nanotubular structure as a study group and polished ceramic titanium oxide as a control, obtaining good results for stem cell differentiation on osteoblasts and stating that the nanotubular surfaces promoted cell differentiation and proliferation, whilst those in the control group suppressed it. This demonstrates that differentiation depends on the architecture of surfaces and their chemistry, being key for regulating cell behavior [28]. If we compare these statements with the results obtained in our study, we agree that the presence of titanium oxide improves cell proliferation.

In a study where bone marrow stem cells were used, the study group (titanium oxide with nanotubular structure obtained by micro-oxidation from an electrolytic solution) showed significant improvements compared to the control group (pure Ti) in the cultures, since it obtained greater adhesion and proliferation without any toxicity. Furthermore, the authors stated that if the sample is reduced electrochemically in saline solution, even better results can be obtained [29]. In our results, none of the samples studied showed signs of cellular toxicity.

Pérez et al. compared nanostructural titanium oxide surfaces with smooth surfaces. On the smooth ones, there was greater cell adhesion and in the ones with oxide, there was less adhesion, although this is focal. This leads to greater differentiation of bone marrow mother stem cells due to the fact that cell movement is greater in this type of surface [57]. If we extrapolate this to our study, we can deduce that greater cell growth happens in the samples modified with titanium oxide pillar array structures could be caused by the presence of nanotubules, which induces greater stress and cell mobilization.

Lv et al. studied the effect of anodized titanium oxide nanotubular surfaces on the differentiation of stem cells from adipose tissue and they compared it to surfaces subjected to sand blasting and double acid etching and machined. Nanotubular surfaces produced greater osteoinduction, being statistically significant for the first two weeks of surface-stem cell contact. Furthermore, they compared 70-nm nanotubes with others measuring 50 and 100 nm in diameter, and the 70-nm ones produced greater osteoinduction. Cell adhesion was greater on nanotubular surfaces, although there were no significant differences with respect to cell proliferation. For the first month, bone formation was greater on nanotubular surfaces, in a statistically significant way [58]. This agrees with our results in the sense that the presence of titanium oxide improved cell growth rate on sand-blasted and acid etched surfaces.

In other studies, cell differentiation was also achieved on titanium oxide surfaces from mice neural stem cells [59]. Kaitainen et al. obtain similar results to those of our study in terms of cell proliferation with 150 nm nanotubes [60].

Other authors stated that surfaces with titanium oxide nanoparticles produced quicker differentiation than in the control groups [61]. This supports the hypothesis that titanium dioxide nanotubular structures are capable of modulating the osteogenic function via mechanotransduction signals.

Pullisaar et al. demonstrated that simvastatin placed on titanium dioxide surfaces produced improvements in gene expression related to osteoinduction and bone tissue formation, if we compare the results with control groups [62,63]. This leads us to consider that perhaps better results would have been obtained on the treated samples of our study if instead of sand blasting them and etching them, we had added simvastatin to them together with the layer of titanium oxide.

The simple fact of being able to have an effect on gene expression related to osteinduction, differentiation and proliferation is encouraging news in the field of oral implantology. All this leads us to think that surfaces modified nanometrically with titanium oxide are capable of improving osteointegration, with significant differences in the first month after implantation. All the current studies confirmed the biocompatibility of this type of surface, its genotoxic capability being controversial. Furthermore, the use of drugs on these types of surfaces is something which has worked in in vitro tests, being helpful in the near future for treatment with implants in patients who are in difficult situations.

All this could be improved with the superhydrophilicity promoted by irradiation with UV light. Our material has a certain optical capacity and no toxicity, so it could be a good candidate for more advanced studies.

## 5. Conclusions

Coatings with titanium oxide were biocompatible in all the samples studied and stimulated greater cell proliferation on grade IV and V titanium surfaces treated with sand blasting and double acid etching and on untreated grade IV surfaces, although this did not occur on untreated grade V titanium surfaces. Nanocolumnar titanium dioxide coatings increased the roughness of grade V surfaces (treated and untreated) and untreated grade IV ones. Titanium oxide-treated surfaces photoactivated by UV irradiation became temporarily superhydrophilic, which supports what other authors have indicated in their studies on the capability of titanium oxide to increase plasma absorbance, thus improving its biocompatibility.

Titanium oxide coatings make aluminum disappeared from the surface and increased oxygen concentration on it. Titanium oxide induced the appearance of calcium on the surface of the treated grade IV and V titanium samples. On the other hand, titanium oxide was conformalon the surface of the implants, forming nanocolumns, which increased plasma and protein adherence, aspects related to an improvement in osteointegration in the initial phases. All this could have interesting applications during osteointegration, increasing calcium phosphate production during this process.

## Figures and Tables

**Table 1:** Nomenclature of the original and modified samples used in the study. (Gr V: Grade V; Gr IV: Grade IV; T: Treated SLA; NT: Non-treated SLA; SI: Treated with plasma generated by an external SLAN-1 microwave source).

**Sample**	**Gr IV NT**	**Gr IV T**	**Gr V NT**	**Gr V T**
Full name	Grade IV–Non treated SLA	Grade IV–Treated SLA	Grade V–Non treated SLA	Grade V–Treated SLA
**Sample**	**Gr IV NT SI**	**Gr IV T SI**	**Gr V NT SI**	**Gr V T SI**
Full name	Grade IV–Non treated SLA/plus treated SLAN-1	Grade IV–Treated/plus treated SLAN-1	Grade V–Non treated/plus treated SLAN-1	Grade V–Treated/plus treated SLAN-1

**Table 2:** Surface atomic percentages determined by XPS found in the non-TiO_2_-coated samples. (GR IV: Titanium grade IV; GR V: Titanium grade V; T: SLA treated; NT: SLA non-treated).

Sample	Gr IV NT	Gr IV T	Gr V NT	Gr V T
%Ti	16.9	21.5	11.4	17.8
%O	46.2	47.0	37.4	44.7
%C	31.6	31.4	43.5	33.5
%Ca	0.2	0.3	0.7	0.1
%Al	-	-	2.4	3.4
%Si	5.0	-	1.0	-
%P	-	-	1.1	-
%N	-	-	1.9	-
%S	-	-	0.5	-
%Na	0.1	-	0.1	-
%F	-	-	-	0.6
O/Ti	2.8	2.2	3.2	2.5
C/TI	1.9	1.5	3.8	1.9

**Table 3:** Surface atomic percentages determined by XPS of the untreated grade IV, treated grade IV, untreated grade V and treated grade V with TiO_2_ coating samples. (GR IV: Titanium grade IV; GR V: Titanium grade V; T: SLA treated; NT: SLA non-treated; SI: Treated with TiO_2_ film).

Sample	Gr IV NT SI	Gr IV T SI	Gr V NT SI	Gr V T SI
%Ti	18.2	9.3	19.5	18.9
%O	44.4	25.4	45.4	44.0
%C	37.4	64.4	35.1	36.9
%Ca	-	0.9	-	0.2
O/Ti	2.4	2.7	2.3	2.3
C/Ti	2.1	6.9	1.8	2.0

**Table 4:** Roughness parameters of the original samples (GR IV: Titanium grade IV; GR V: Titanium grade V; T: SLA treated; NT: SLA non-treated).

Sample	Ra (µm)	Rq (µm)	Rz (µm)
Gr IV NT	0.193	0.241	1.103
Gr IV T	1.240	1.675	11.442
Gr V NT	0.103	0.132	0.822
Gr V T	1.137	1.394	6.978

**Table 5:** Roughness parameters of the samples treated with TiO_2_ film (GR IV: Titanium grade IV; GR V: Titanium grade V; T: SLA treated; NT: SLA non-treated; SI: treated with TiO_2_ film).

Sample	Ra (µm)	Rq (µm)	Rz (µm)
Gr IV NT SI	0.101	0.132	0.922
Gr IV T SI	1.140	1.434	7.689
Gr V NT SI	0.122	0.155	0.860
Gr V T SI	1.203	1.461	7.290

**Table 6:** Atomic percentages of the elements of the untreated with TiO_2_ film and non-treated SLA grade IV and V samples and untreated with TiO_2_ film and treated SLA grade IV and V samples. (GR IV: Titanium grade IV; GR V: Titanium grade V; T: SLA treated; NT: SLA non-treated).

Sample	Gr IV NT	Gr IV T	Gr V NT	Gr V T
Norm. At. %Ti	89.8	95.6	63.0	78.3
Norm. At. %O	7.0	1.4	28.6	11.9
Norm. At. %C	2.7	1.9	1.8	2.3
Norm. At. %Al	0.5	1.1	6.6	7.5

**Table 7:** Atomic percentages of the elements of the treated with TiO_2_ film and non-treated SLA grade IV and V samples and treated with TiO_2_ film and treated SLA grade IV and V samples. (GR IV: Titanium grade IV; GR V: Titanium grade V; T: SLA treated; NT: SLA non-treated; SI: Treated with TiO_2_ film).

Sample	Gr IV NT SI	Gr IV T SI	Gr V NT SI	Gr V T SI
Norm. At. % Ti	77.3	64.9	74.4	75.1
Norm. At. % O	22.5	33.9	25.3	24.6
Norm. At. % C	0.2	1.2	0.3	0.3
Norm. At. % Al	-	-	-	-

**Table 8:** Surface tensions and contact angles of the original samples. (GR IV: Titanium grade IV; GR V: Titanium grade V; T: SLA treated; NT: SLA non-treated).

Sample	σSD (mN/m)	ΣsP (mN/m)	σS (mN/m)	θ Water	θ Diodomethane	Rw	Ideal θ Water
Gr IV NT	27.82	9.90	37.72	72.9	45.6	1.044	73.6
Gr IV T	73.30	9.82	83.12	121.1	23.2	2.360	102.6
Gr V NT	34.52	3.23	38.75	83.9	44.0	1.011	83.9
Gr V T	38.60	0.77	39.37	112.1	57.7	-	-
Si (100)	26.52	39.18	65.70	27.3	10.2	-	-

**Table 9:** Surface tensions and contact angles of the samples with TiO_2_ coating. (GR IV: Titanium grade IV; GR V: Titanium grade V; T: SLA treated; NT: SLA non-treated; SI: Treated with TiO_2_ film).

Sample	σSD (mN/m)	ΣsP(mN/m)	σS(mN/m)	θ Water	θ Diodomethane	Rw	θ Ideal Water
Gr IV NT SI	48.30	0.83	49.13	106.7	43.6	1.062	105.7
Gr IV T SI	45.19	7.41	52.60	132.2	61.3	1.542	115.8
Gr V NT SI	48.10	1.06	49.16	108.2	44.9	1.058	107.2
Gr V T SI	62.07	14.02	76.09	136.4	47.3	-	-
Si (100) + SI	53.99	2.05	56.04	109.8	39.2	-	-

**Table 10:** Initial and final water contact angles (on applying UV irradiation) of the treated and untreated grade IV and grade V samples. (GR IV: Titanium grade IV; GR V: Titanium grade V; T: SLA treated; NT: SLA non-treated).

Sample	Gr IV NT	Gr IV T	Gr V NT	Gr V T
θ initial	80	117	84	114
θ final	27	13	16	21
Time (s)	8298	5760	16,900	9400

**Table 11:** Initial and final water contact angles (on applying UV irradiation) of the grade IV and grade V samples with TiO_2_ film. (GR IV: Titanium grade IV; GR V: Titanium grade V; T: SLA treated; NT: SLA non-treated; SI: Treated with TiO2 film).

Sample	Gr IV NT SI	Gr IV T SI	Gr V NT SI	Gr V T SI
θ initial	103	135	104	136,4
θ final	21	108	20	24
Time (s)	7200	7920	8640	5760

**Table 12:** Absorbance and number of cells arising from the result on the samples studied in cell culture.

**Sample**	**Gr IV NT**	**Gr IV T**	**Gr V NT**	**Gr V T**
Absorbance	1.30	0.81	1.28	0.80
Number of adhered cells (×10^3^)	11.60	7.23	11.42	7.14
**Sample**	**Gr IV NT SI**	**Gr IV T SI**	**Gr V NT SI**	**Gr V T SI**
Absorbance	1.31	0.85	1.12	0.92
Number of adhered cells (×10^3^)	11.69	7.58	10.01	8.21
